# Editorial: Discourse, conversation and argumentation: theoretical perspectives and innovative empirical studies, volume III

**DOI:** 10.3389/fpsyg.2024.1505219

**Published:** 2024-10-21

**Authors:** Antonio Bova, Carlo Galimberti, Francesco Arcidiacono, Lise Haddouk

**Affiliations:** ^1^Department of Psychology, Catholic University of the Sacred Heart, Milan, Italy; ^2^PSICOM–Centro Studi e Ricerche di Psicologia della Comunicazione, Catholic University of the Sacred Heart, Milan, Italy; ^3^Research Department, Haute Ecole Pédagogique—Cantons de Berne Francophone, Biel/Bienne, Switzerland; ^4^Department of Psychology, Université de Rouen, Mont-Saint-Aignan, France

**Keywords:** argumentation, communicative interactions, conversation, discourse, psychology

Despite the fundamental role that discursive, conversational, and argumentative interactions play in daily life, psychological research has yet to establish a comprehensive framework for understanding these forms of communication. Current investigations into discourse, conversation, and argumentation are fragmented across distinct research communities, often siloed within separate disciplines, resulting in limited interdisciplinary dialogue. This third volume of the Research Topic, “*Discourse, conversation and argumentation: theoretical perspectives and innovative empirical studies*,” seeks to bridge this gap by offering an integrated platform for advancing theoretical and empirical inquiry into communicative processes from a psychological lens. In particular, this volume presents groundbreaking research that delves into the dynamics of these interactions across individual, group, and institutional contexts, while emphasizing recent methodological innovations in their study. By consolidating contributions from diverse international perspectives, this Research Topic fosters a rich exchange of ideas, offering a state-of-the-art synthesis of psychological research on communication.

Contributors to this Research Topic bring fresh insights, grounded in rigorous empirical work, that span the spectrum of discursive, conversational, and argumentative interaction. The range of perspectives presented here not only reflects the field's vibrancy but also signals a promising convergence of approaches that transcend disciplinary boundaries. By assembling such a distinguished group of scholars, this volume arrives at a critical juncture, providing valuable guidance for those navigating the complexities of specialized research in this field. We are confident that the contributions within this volume will inspire and challenge a broad academic audience, driving the integration of psychological research on communicative interactions and promoting future interdisciplinary collaboration.

Convertini and Luciani delve into the intricate social co-construction of psychologist and patient roles across various contextual domains, offering a profound exploration of how these dynamics are shaped. Leveraging the Argumentum Model of Topics, their research underscores the pivotal role of implicit contextual premises in perpetuating mental health stigma. Their findings reveal that in well-defined institutional settings, institutional norms tend to prevail, whereas more informal domains allow individual perspectives to take precedence. This study emphasizes the urgent need to dismantle outdated premises in order to reduce stigma and enhance mental health awareness.

Liu M. et al. contribute a corpus-assisted discourse analysis of China's official diplomatic speeches, highlighting themes of cooperation, development, and peace, with an emphasis on President Xi Jinping's leadership. Their analysis uncovers how China's diplomatic discourse frames its international relations and shapes its global image, shedding light on the ideological underpinnings of China's foreign policy rhetoric.

Wang examines the role of teacher care behaviors in reducing EFL (English as a Foreign Language) learners' anxiety. Through a chain mediation model, Wang identifies student engagement and learning strategies as key factors in mitigating anxiety, offering educators valuable insights into creating supportive and effective teaching environments that enhance learning outcomes in EFL contexts.

Wu et al. provide a comparative analysis of self-praise strategies on English and Chinese social media platforms, revealing distinct cultural patterns. On Twitter, users tend to employ more explicit self-praise strategies, whereas Weibo users favor implicit expressions. The study explores how factors such as appearance and virtues differ across platforms and the psychological and commercial motivations that drive self-praise. This research deepens our understanding of cross-cultural differences in online self-presentation.

Xu and Ge focus on same-turn self-repair in Chinese civil courtroom interactions, illustrating how participants strategically use language to manage credibility and adjust their epistemic stance. Their findings highlight the importance of repair mechanisms in navigating complex legal discourse, offering critical insights into communication strategies within institutional settings.

Ni applies role-theoretic discourse analysis to Germany's parliamentary debates on military operations in Afghanistan. Identifying five key topoi—necessity, obligation, self-interest, capability, and solution—the study provides an in-depth analysis of how these argumentative themes shape Germany's evolving security policy. Through this lens, the research offers a fresh perspective on Germany's shifting role as a global actor in the international arena.

Lee et al. investigate age-related changes in speech production using eye-tracking during picture description tasks. Their results indicate that older adults demonstrate reduced linguistic productivity and cognitive processing efficiency compared to younger individuals. These findings offer important implications for understanding how aging impacts communication and cognitive function.

Hosokawa and Kitagami explore the cognitive processes involved in visuospatial perspective-taking during narrative comprehension. Their research reveals that shifting perspectives between characters significantly increases cognitive load, highlighting how narrative complexity interacts with individual differences in working memory to influence comprehension.

Zhuo introduces a pioneering approach to affective pragmatics through the framework of Darwinian Biolinguistics. Zhuo's study investigates how biological factors, particularly those related to emotional processing in the brain, shape the way language is used and interpreted. This interdisciplinary research opens new pathways for understanding the intersection of biology, emotion, and communication.

Liu L. et al. present a novel high-frequency sense list designed to aid language learners in prioritizing the most common meanings of words. By semantically annotating large corpora, the researchers offer a powerful tool for improving vocabulary acquisition, particularly for beginners, thus providing more efficient strategies for language learning.

These studies collectively offer significant contributions to psychological and linguistic research, enhancing our understanding of communicative interactions across a variety of contexts. From the social stigmas of mental health to the intricacies of narrative comprehension, this body of work expands the boundaries of psychological science and provides fresh insights into the complexities of human communication.

